# Influence of natural variation in berry size on the volatile profiles of *Vitis vinifera* L. cv. Merlot and Cabernet Gernischt grapes

**DOI:** 10.1371/journal.pone.0201374

**Published:** 2018-09-19

**Authors:** Sha Xie, Yonghong Tang, Peng Wang, Changzheng Song, Bingbing Duan, Zhenwen Zhang, Jiangfei Meng

**Affiliations:** 1 College of Enology, Northwest A & F University, Yangling, Shaanxi, China; 2 Zhengzhou Fruit Research Institute, Chinese Academy of Agricultural Sciences, Zhengzhou, China; 3 Shaanxi Engineering Research Center for Viti-Viniculture, Yangling, Shaanxi, China; Universidade de Lisboa Instituto Superior de Agronomia, PORTUGAL

## Abstract

This study was conducted during the 2014 and 2015 vintages on *Vitis vinifera* L. cv. Merlot and Cabernet Gernischt to investigate whether natural variation in berry size could affect grape aromatic compounds. Grape berries were separated into three size categories based on their diameter: small, middle and large. The results showed that berry size exerted a significant influence on the volatile profiles of both winegrape varieties. Hierarchical clustering analysis demonstrated that the volatile profiles of middle berries were different from those of large and small berries. Middle berries had the greatest abundance of aroma compounds, followed by small and large berries. Especially, C6/C9 compounds, norisoprenoids, terpenoids showed markedly different concentrations among differently sized Merlot berries and C6/C9 compounds, terpenoids among differently sized Cabernet Gernischt berries. Middle berries of both grape varieties may possess the greatest intensity of fresh-green, fruity and floral aromas due to the high odour activity values (OAVs) of decanal, hexanal, (E)-2-hexenal, (E)-β-damascenone and β-ionone in middle sizes of Merlot berries and the high OAVs of (E)-2-hexenal and (E)-β-damascenone in middle sizes of Cabernet Gernischt berries. This knowledge could be important for winemakers to conduct targeted berry sorting, thereby improving the aromatic quality of grapes.

## Introduction

The production of uniform parcels of fruit is essential for grapevine cultivation. However, considerable asynchrony exists between grape berries within a bunch and between bunches on a vine due to the influence of light interception, temperature, nutrient availability, and drought stress, among other factors [1]. Targeted berry sorting has been proposed as a method to minimize berry heterogeneity, thus increasing wine quality [2]. Previous studies on berry sorting have focused on berry size and colour [3, 4], berry density [1], berries at different heights on vines [5], and berries from different cluster positions [6], among which berry diameter is an easily measured and highly variable parameter in vineyards. In fact, recently optical berry sorting machines capable of accurately assessing the berry size of winegrapes, have been developed [3].

Berry size, related to the skin-to-juice and surface-to-volume ratios, is an important quality trait of winegrapes. Until now there have been no consistent conclusions regarding the relationship between berry size and berry compositions: Walker et al [7] reported that berry size did not affect the quality of wine; while Rolle et al [1] and Wong et al [8] showed that the smaller berries contained a higher concentration of many skin-located compounds, thus having higher quality characteristics. Besides, these studies mainly focused on phenolic substances, the available results of the relationship between berry size and volatile compounds are scarce.

Grape-originated aroma makes an important contribution to final wine flavor [9]. Volatile compounds of grapes are sensitive to microclimate, and many studies have found internal variability among clusters from the same vine and among berries within the same cluster for different physical and chemical parameters [10–12]. Even berries from the tip and shoulder of the same cluster exhibited different aroma profiles [6]. Recently, Friedel et al [3] found that wines from differently sized berries of *Vitis vinifera* L. cv. Riesling had a pronounced difference in aroma compounds. Moreover, Wong et al [8] found that there were transcriptome changes involving aroma pathways between Merlot (*Vitis vinifera* L. cv.) berries of different sizes, which indicated there might be aromatic differences between differently sized berries. However, research concerning the comprehensive volatile profiles of differently sized grape berries is limited.

Cabernet Gernischt and Merlot (*Vitis vinifera* L. cv.) are the most important red wine cultivars in China [13]. In 2012, Zhong et al established that Cabernet Gernischt is in fact Carmenère, the old Bordeaux variety now so common in Chile [14]. In Ningxia Autonomous Region, a wine production base in China, Merlot is commonly blended with Cabernet Gernischt to add the spice aromas to Cabernet Gernischt wine. However, there are few works published on volatiles of Cabernet Gernischt and Merlot berries. To the best of our knowledge, only Fan et al reported that the most intense odorants in Cabernet Gernischt and Merlot berries were β-damascenone, hexanal, (Z)-3-hexen-1-ol, (E,Z)-2,6-nonadienal and β-ionone [13], which indicated that these two berries have fresh, fruit flavors and potentially leafy, vegetal notes. Furthermore, the scientific literature on the volatiles of differently-sized Merlot and Cabernet Gernischt berries is even more limited.

Most of the published research related to berry size was associated with certain pruning treatments or deficit irrigation strategies [4, 15], and thus, it is difficult to determine whether compositional differences between berries of different sizes are due to berry size per se or treatment effects. This study was conducted over two seasons on *Vitis vinifera* L. cv. Merlot and Cabernet Gernischt, with the aim of investigating the relationship between the natural variations in berry size and grape chemical composition, especially aroma compounds. This paper will assist winemakers in conducting targeted berry sorting according to berry size, thus increasing the aromatic quality of grapes.

## Materials and methods

### Vineyard description and meteorological data acquisition

Merlot and Cabernet Gernischt winegrape cultivars (*Vitis vinifera* L. cv.) were harvested in the 2014 and 2015 vintages from Yuquanying (YQY) Farm of Ningxia Autonomous Region, China (38° 28' N; 106° 16' E). This farm belongs to private vineyard. No specific permissions were required for the experimental locations. The field studies did not involve endangered or protected species. This region has a mid-temperate continental monsoon climate with an annual mean temperature of 9.4°C, an annual average rainfall of 193.4 mm, 3000 annual sunshine hours and 185 frost-free days. Both selected winegrape cultivars were more than 5-year-old own rooted plants and the vineyards were managed according to the standard agronomic practices of the region.

Geographic coordinates (latitude, longitude) were determined using Google Earth (Google Inc, USA). Climate values and meteorological data (mean temperature and rainfall) were obtained from the China Meteorological Data Sharing Service System (http://cdc.cma.gov.cn/home.do). The average temperature and rainfall in July, August and September of 2014 and 2015 are shown in **S1 Fig**.

### Berry sampling and size segregation

Samples were collected at commercial harvest in 2014 and 2015 by monitoring TSS. Four biological replicates (10 kg of grapes per replicate) were collected, following the methods described by Falginella et al [16]. The berries of each replicate were randomly selected by hand from more than 60 different grapevines on both sides of the canopy. Each replicate was collected from a different plot. All the samples were immediately classified according to their diameter using sieves on the basis of preliminary observations. Merlot berries were divided into three size categories, small berries (<12 mm), middle berries (12–14 mm) and large berries (>14 mm), using 2 sieves with mesh diameters of 12 mm and 14 mm. Cabernet Gernischt berries were separated into small berry (<14 mm), middle berry (14–15 mm) and large berry groups (>15 mm) using 2 sieves with mesh diameter of 14 mm and15 mm. All the sorted berries were frozen in liquid nitrogen and stored at -80°C for analysis.

### Berry physical characteristics and technological ripeness parameters

For each sample set and class, one subsample of 100 sorted berries was weighed, and then the skin, flesh, and seeds were separated from the 100 frozen berries using a scalpel. Subsequently, the skin mass, seed mass and seed numbers of the 100 berries were determined.

Another subsample of 100 sorted berries was manually juiced to determine the technological ripeness parameters. TSS was measured using a TD-45 digital refractometer (Zhejiang Top Instrument Co., Ltd., Hangzhou, China). The pH measurement was performed with a PB-10 pH meter (Sartorius, Gottingen, Germany).

### Extraction and determination of volatile compounds

#### Isolation of aroma compounds

For each sample set and class, another subsample of 100 g sorted berries was pitted, ground and blended with 1 g of polyvinylpolypyrrolidone (PVPP). The flesh was macerated at 4°C for 240 min and then centrifuged at 8000 rpm at 4°C for 15 min to obtain clear juice. Five millilitres of clear juice, 1 g of NaCl and 10 μL of 4-methyl-2-pentanol (1.039 mg/mL water, internal standard) were blended in a 15-mL vial containing a magnetic stirrer. The vial was tightly capped with a PTFE-silicone septum. Volatile compounds were extracted using headspace solid-phase microextraction (HS-SPME) with a 2 cm DVB/CAR/ PDMS 50/30 μm SPME fibre (Supelco, Bellefonte, PA., USA) on a CTC CombiPAL autosampler (CTC Analytics, Zwingen, Switzerland). The SPME fibre was conditioned at 250°C for 1 h prior to extraction. After being equilibrated at 40°C for 30 min under stirring at 500 rpm, the samples were extracted with the pre-conditioned SPME fibre at 40°C for 30 min under continued heating and agitation. Subsequently, the fibre was immediately desorbed in the GC injector for 8 min at 250°C [17, 18].

#### GC–MS analysis

The separation and identification of the volatile compounds were performed on an Agilent 6890 GC with an HP-INNOWAX capillary column (60 m×0.25 mm ×0.25 μm, J&W Scientific, Folsom, CA, USA) coupled to an Agilent 5975 MS. The GC–MS temperature conditions in this study were based on previous work by Wu et al [19]. Helium, the carrier gas, flowed at a constant rate of 1 mL/min in splitless mode. The injection temperature was set to 250°C. The oven temperature was programmed to hold at 50°C for 1 min and then increase to 220°C at 3°C /min, where it was held for 5 min. The MS conditions were as follows: electron ionization (EI) mode at 70 eV; 230°C ion source temperature; 280°C MS transfer line temperature; scan from m/z 20 to 350.

#### Qualitative and quantitative analysis

The identification of the volatile compounds was based on retention indices (RIs) of reference standards and mass spectra matching in the standard NIST 11 MS database. When reference standards were not available, volatile compounds were tentatively identified by comparing their mass spectra with the NIST 11 MS database and the RIs reported in previous literature or RIs sourced in the NIST Standard Reference Database (http://webbook.nist.gov/chemistry/) [20]. The quantification of the volatile compounds followed the internal standard-standard curve method, and 4-methyl-2-pentanol (1.039 mg/mL water) was used as the internal standard. The simulated juice solution was prepared according to the average concentration of sugars and acids in the samples. The standard volatile components dissolved in ethanol (HPLC quality) were added in the simulated juice solution, and the mixture was then diluted successively into fifteen levels with the simulated juice solution. The aroma standards of each level were extracted and analysed under the same condition as the grape sample to obtain calibration curves, all presenting coefficients above 98%. In addition, volatile compounds without calibration curves were quantified with standards that had the same functional groups and/or similar numbers of C atoms. The detailed quantification information is listed in **S1 Table**.

### Odour activity values (OAVs)

To evaluate the contribution of volatile compounds to grape berry aroma, OAVs were determined in this study. OAVs were calculated as the ratio between an individual compound concentration and the perception threshold from aqueous solution [21].

### Statistical analysis

SPSS 20.0 (IBM, Armonk, NY, USA) was used to analyse the statistical parameters of the volatile compounds. One-way analysis of variance (ANOVA) and Tukey’s HSD test were conducted to detect significant differences in the volatile compounds of differently sized berries. Hierarchical clustering analysis was performed using MetaboAnalyst 2.0 (http://www.metaboanalyst.ca/) through the ‘Statistical Analysis’ interface, and data were normalized using ‘Autoscaling’ (mean-centred and divided by the standard deviation of each variable) in the MetaboAnalyst program. The rest of the plots were prepared using OriginPro 8.5 (OriginLab Corporation, Northampton, MA, USA).

## Results and discussion

### Distribution of two grape varieties (*Vitis vinifera* L. cv.) in different size classes

**Fig 1:**
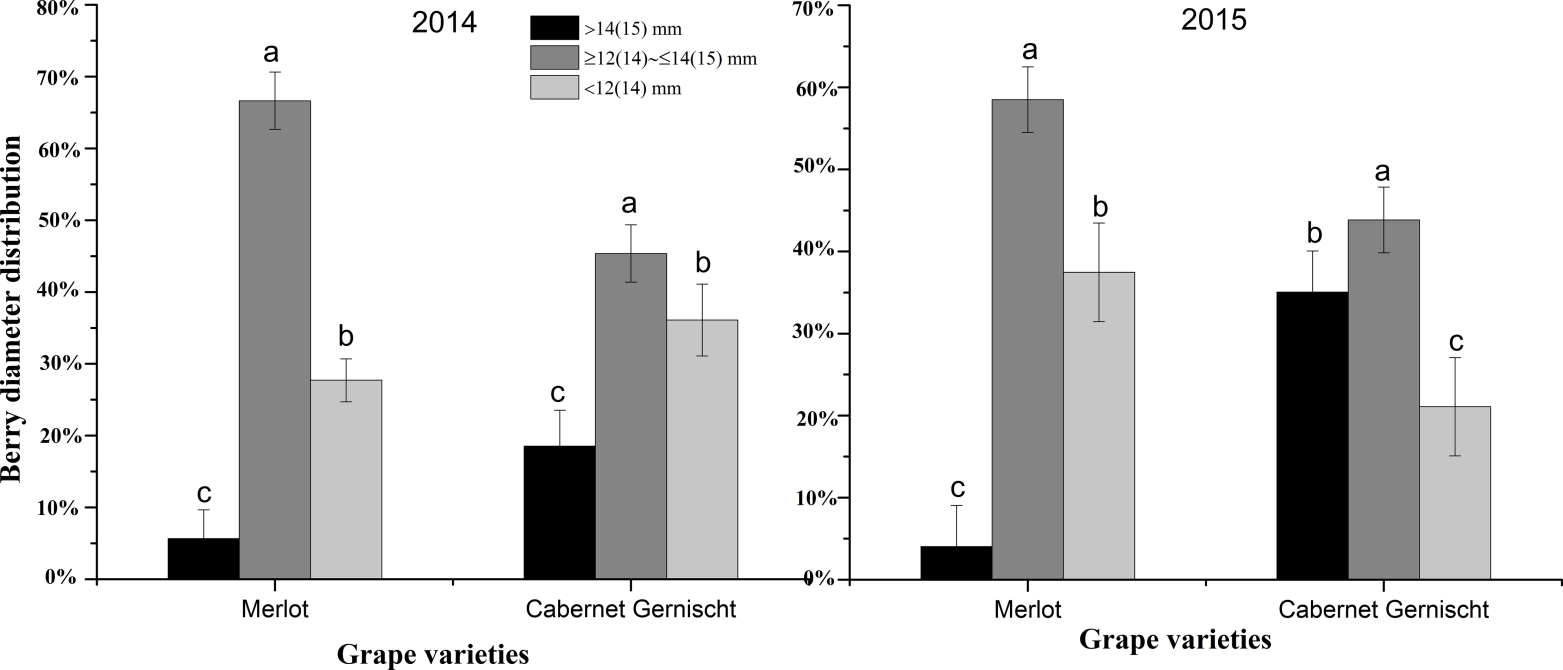
Distribution of two grape varieties (*Vitis vinifera* L. cv.) (%) in different size classes at the commercial harvests in 2014 and 2015. Size classification of Merlot berry: small berries (<12 mm); middle berries (12–14 mm); large berries (>14 mm). Size classification of Cabernet Gernischt berry: small berries (<14 mm); middle berries (14–15 mm); large berries (>15 mm). Tukey’s HSD test, different letters in each grape variety indicate significant differences at P < 0.05.

Berries from both grape varieties *(Vitis vinifera* L. cv.) were segregated into 3 size categories. **Fig 1** shows that the majority of the berries appeared in the middle diameter group (12(14)–14(15) mm) in the successive two years, consistent with previous studies which reported that berry size distribution followed a standard Gaussian curve [1]. The percentages of diameter distribution of Merlot berries were similar in both years, while Cabernet Gernischt berries showed higher percentage of large berry distribution and lower percentage of small berry distribution in 2015 than that in 2014. This might be attributed to in-field grape variability due to climatic variables (sunlight, temperature, water status, etc.).

### Technological ripeness parameters

**Table 1** shows the technological ripeness parameters of two grape varieties (*Vitis vinifera* L. cv.) at the commercial harvests in 2014 and 2015.The TSS of all berries were above 20 Brix in both vintages, which suggested that grapes from both vintages achieved acceptable maturity for any berry diameter evaluated [18]. Within all berry diameter classes, TSS increased with the decreased berry size in the 2014 vintage, in agreement with the study of Roby et al [4]. However, in the 2015 vintage, the middle berries had the highest TSS compared with small and large berries. Previous research reported that TSS showed no consistent trend with berry size [7] or decreased with increasing berry size [4]. In our study, the effect of berry size on TSS was not consistent over the two seasons, indicating that there was a seasonal effect on TSS [22]. Overall, berries from 2015 exhibited a higher maturity degree than those from 2014 did, which may be due to the higher average temperature and lower rainfall in 2015(**S1 Fig)** in that temperature and rainfall could affect grape ripeness [23].The small berries of both varieties had the highest maturity degree in 2014 but had the lowest maturity degree in 2015, indicating the seasonal difference exerted a great influence on small berries. The differences of pH between differently sized berries were not consistent, in line with previous study [1].

**Table 1:** Technological ripeness parameters of two grape varieties (*Vitis vinifera* L. cv.) in different size classes at the commercial harvests in 2014 and 2015.

The variety of grapes	Diameter class	2014 Vintage		2015 Vintage	
		Total soluble solids (%)	pH	Total Soluble solids (%)	pH
Merlot	Large	20.20 ± 0.38b^1^	3.67 ± 0.01b	25.10 ± 0.10b	3.75 ± 0.01a
	Middle	21.90 ± 0.24a	3.81 ± 0.01a	25.55 ± 0.17a	3.39 ± 0.01b
	Small	23.00 ± 0.48a	3.80 ± 0.01a	24.10 ± 0.10c	3.31 ± 0.01c
Cabernet Gernischt	Large	20.50 ± 0.29c	3.85 ± 0.01ab	23.00 ± 0.15b	4.73 ± 0.04a
	Middle	20.70 ± 0.15b	3.82 ± 0.01b	23.30 ± 0.15a	4.19 ± 0.00c
	Small	21.25 ± 0.22a	3.87 ± 0.01a	22.35 ± 0.22c	4.47 ± 0.00b

^1^ Tukey’s HSD test, different letters indicate significant differences at P < 0.05.

### Berry physical characteristics

The small berries had significantly higher skin-to-berry mass ratios, with the exception of the Cabernet Gernischt in 2015 (**Table 2**). Therefore, these small berries might contain a higher concentration of grape skin compounds [24]. The seed-to-berry mass ratio, single seed weight and seed number per berry increased with increasing berry size. This relationship between berry size and seed content might result from the growth regulators produced by seeds [25] and/or from the hormone-related genes which have shown different expression in differently sized berries [8].

**Table 2:** Physical characteristics of differently sized *Vitis vinifera* L. cv. Merlot and Cabernet Gernischt berries in 2014 and 2015.

		2014 Vintage				2015 Vintage			
The variety of grape	Diameter class	Skin /Berry mass ratio (%)	Seed /Berry mass ratio (%)	Single seed weight (g)	Seed number per berry	Skin /Berry mass ratio (%)	Seed /Berry mass ratio (%)	Single seed weight (g)	Seed number per berry
Merlot	Large	7.02 ± 0.46b^1^	7.38 ± 0.45a	0.14 ± 0.006a	2.75 ± 0.18a	7.60 ± 0.53c	7.05 ± 0.37a	0.13 ± 0.007a	2.45 ± 0.12a
	Middle	7.27 ± 0.51b	6.76 ± 0.49b	0.09 ± 0.005b	1.94 ± 0.15b	9.81 ± 0.62b	6.45 ± 0.30b	0.08 ± 0.006b	1.80 ± 0.11b
	Small	9.50 ± 0.79a	5.37 ± 0.35c	0.05 ± 0.003c	1.10 ± 0.06c	11.68 ± 0.60a	5.10 ± 0.24c	0.04 ± 0.002c	1.10 ± 0.08c
Cabernet Gernischt	Large	8.93 ± 0.56b	3.37 ± 0.17a	0.08 ± 0.005a	1.94 ± 0.13a	8.19 ± 0.65c	3.60 ± 0.24a	0.08 ± 0.006a	1.60 ± 0.14a
	Middle	7.17 ± 0.47c	3.19 ± 0.15a	0.05 ± 0.003b	1.08 ± 0.05b	8.73 ± 0.70a	3.39 ± 0.21b	0.06 ± 0.004b	1.20 ± 0.12b
	Small	14.07 ± 1.20a	2.88 ± 0.13b	0.04 ± 0.002c	1.06 ± 0.04b	8.57 ± 0.68b	2.85 ± 0.18c	0.04 ± 0.002c	0.95 ± 0.07c

^1^ Tukey’s HSD test, different letters indicate significant differences at P < 0.05.

### Volatile profiles of two grape varieties (*Vitis vinifera* L. cv.) in different sizes

**Fig 2:**
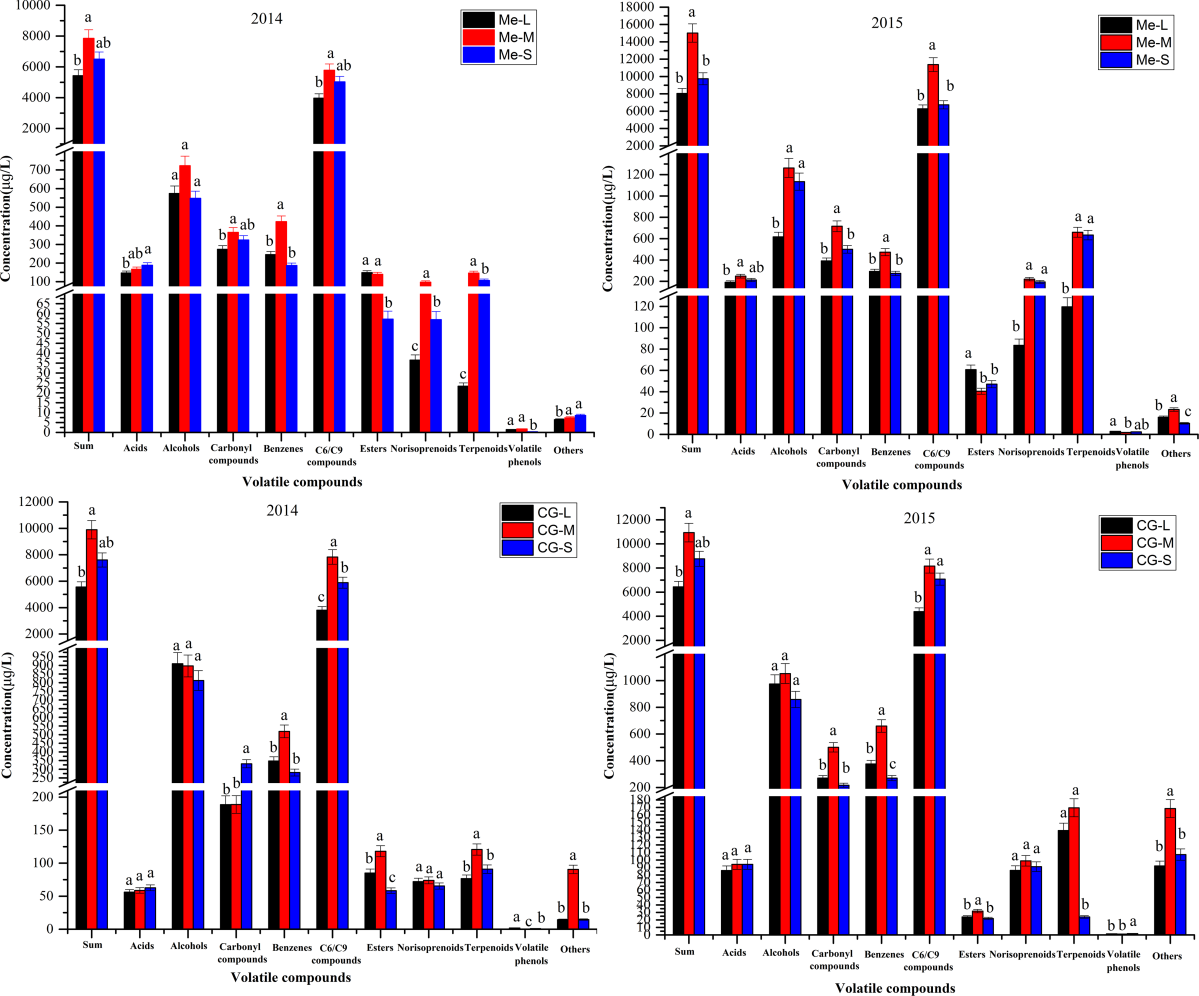
Volatile compounds of differently sized *Vitis vinifera* L. cv. Merlot and Cabernet Gernischt berries in 2014 and 2015. Me-L: Large berries of Merlot; Me-M: Middle berries of Merlot; Me-S: Small berries of Merlot. CG-L: Large berries of Cabernet Gernischt; CG-M: Middle berries of Cabernet Gernischt; CG-S: Small berries of Cabernet Gernischt. Tukey’s HSD test, different letters in each grape variety indicate significant differences at P < 0.05.

Grape-originated aroma plays an important role in potential wine aroma. In this study, a total of 76 and 79 volatile compounds were identified in *Vitis vinifera* L. cv. Merlot and Cabernet Gernischt, respectively (**S2 Table and S3 Table**). The volatile profiles of both cultivars were characterized by the aroma compounds for non-Muscat grape cultivars, with the highest concentration being C6/C9 compounds and the second-highest content being alcohols (**Fig 2**), which was consistent with previous studies [13, 18, 26]. Overall, the volatile compounds of both grape varieties in 2015 were found to have higher total concentrations than that in 2014, which was most likely due to the greater maturity degree of both grape varieties from 2015 than that from 2014 [27]. Though the global volatile contents of both grape varieties from the 2015 vintage were much higher than that from 2014, the effects of berry size on the volatile profiles were consistent in both vintages, with middle berries having the significantly highest total volatile concentrations, followed by small berries and large berries in both years. The C6/C9 compounds, as the predominant components in the both cultivars, showed significant differences among differently sized berries, with significantly higher concentrations in the middle berries of the 2014 and 2015 vintages. In addition, the concentrations of C6/C9 compounds in 2015 were higher than in 2014. That may be because the C6 compounds are derived from the LOX-HPL pathway and less rainfall in 2015 (**S1 Fig**) might increase the transcript abundance of LOX and HPL, thereby higher levels of C6 aldehydes could be produced [28]. Alcohols, the second-most abundant compounds, exhibited no significant concentration differences between differently-sized Cabernet Gernischt berries in both years and Merlot berries in 2014 vintage, while showed markedly higher concentrations in the middle and small berries of Merlot in the 2015 vintage. Although norisoprenoids and terpenoids had low concentrations in Merlot and Cabernet Gernischt berries, they make important contributions to the characteristic varietal aroma due to their extremely low odour thresholds [17]. Norisoprenoids had markedly higher concentrations in middle sizes of Merlot berries compared to that in large berries, whereas showed no significant concentration differences between differently-sized Cabernet Gernischt berries in both years. Terpenoid compounds showed the greatest abundance in middle size of Merlot and Cabernet Gernischt berries in both years. Similar results were found in the study of Friedel et al [3], who reported that middle Riesling berries (12.5–14 mm) had relatively high norisoprenoid and terpenoid concentrations compared to large berries (14–16 mm). Norisoprenoids and terpenoids, as grape-derived volatile compounds, underwent minimal changes during the wine fermentation process [29,30], and thus their concentrations in berries likely influence the final aroma profiles of wines.

**Fig 3:**
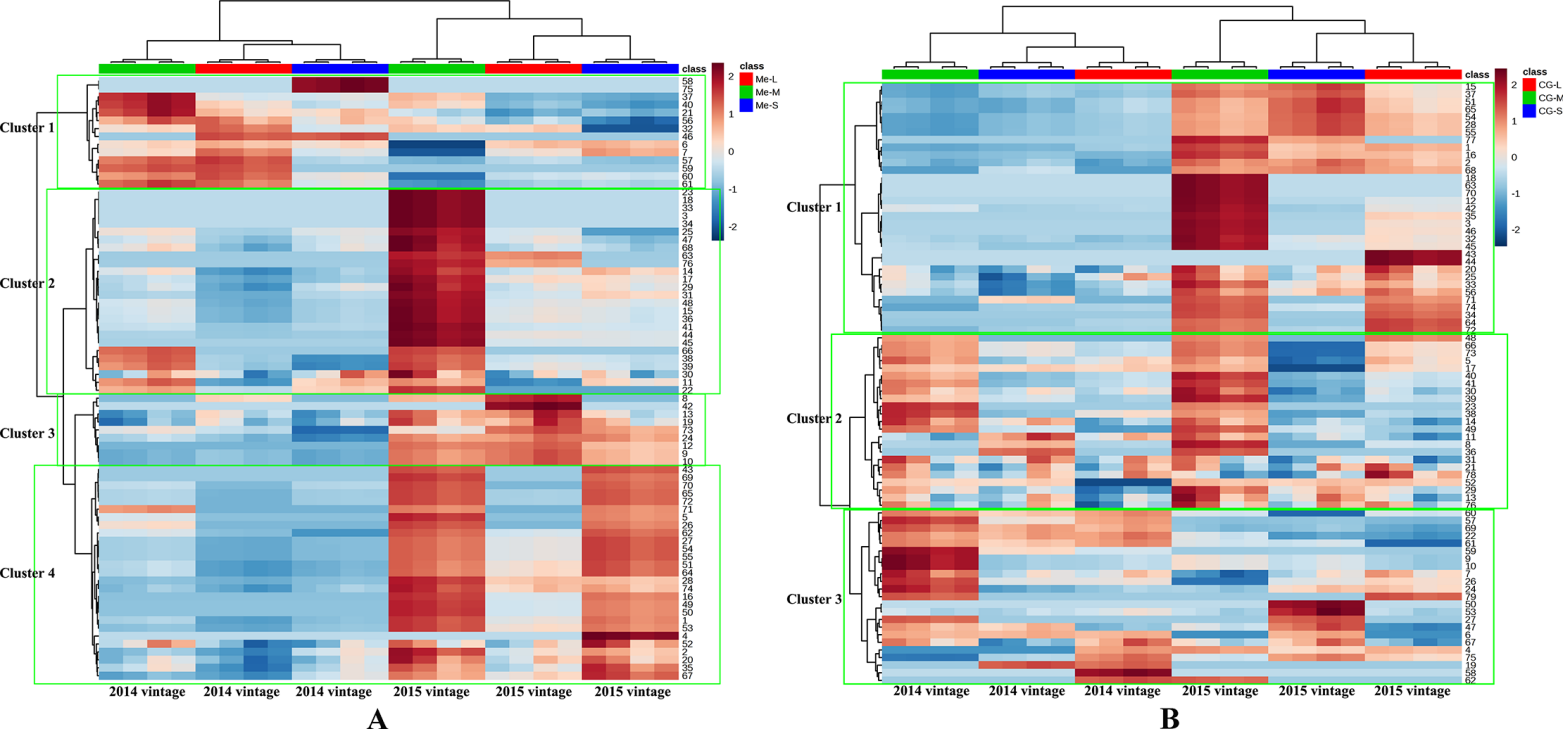
Hierarchical clustering analysis dendrogram for volatile compounds of *Vitis vinifera* L. cv. Merlot and Cabernet Gernischt in different size classes in 2014 and 2015. Me-L: Large berries of Merlot; Me-M: Middle berries of Merlot; Me-S: Small berries of Merlot. CG-L: Large berries of Cabernet Gernischt; CG-M: Middle berries of Cabernet Gernischt; CG-S: Small berries of Cabernet Gernischt. The number in each row in A and B corresponding to each volatile is presented in **S2 Table** and **S3 Table**, respectively.

Hierarchical clustering analysis was applied to evaluate the use of berry size to discriminate different quality berries in terms of profiles of volatile compounds. **Fig 3** shows the hierarchical clustering analysis dendrogram of the volatile compounds of *Vitis vinifera* L. cv. Merlot and Cabernet Gernischt berries sorted by berry size. In this dendrogram, the samples were grouped in terms of their nearness or similarity. A difference between the volatile profiles of differently sized berries was well visualized by a clustering heatmap. As shown in **Fig 3A**, volatile compounds of small and large Merlot berries were grouped into a cluster and differed from the volatiles of middle berries during the 2014 and 2015 seasons, which indicated that the volatile profile of middle sizes of Merlot berries was different from those of large and small berries. Volatile compounds of Merlot berries sorted by berry size could be grouped into four main clusters according to calculated distances. Overall, Cluster 1 represented the compounds that exhibited the higher concentrations in 2014 than that in 2015; Cluster 2, Cluster 3 and Cluster 4 represented the volatiles having higher levels in the 2015 season than that in the 2014 season; Cluster 2 represented the compounds that exhibited the significantly high concentrations in the middle berries; Cluster 3 represented the compounds that had higher concentrations in the large berries than in the small and middle berries; Cluster 4 represented the compounds that were characterized by having much higher concentrations in the small and middle berries than in large berries; Cluster 2 and Cluster 4 were the major components contributing the more aromatic characteristics in the middle berries. They include 2 acids (1, 2), 10 alcohols (3, 4, 5, 11, 14, 15,16, 17,18,20), 13 carbonyl compounds (22,23,25,26,27,28,29,30,31,33,34,35,36),6 benzenes (38,39,41,43,44,45), 9 C6/C9 compounds (47,48,49,50,51,52,53,54,55),2 esters (62,63), 4 norisoprenoids (64,65,66,67) and 5 terpenoids (68,69,70,71,72).

Hierarchical cluster analysis of the volatile compounds of Cabernet Gernischt berries showed that the middle berry group was clearly discernible from the large and small berry groups. Cluster analysis of the heatmap grouped the volatile compounds of all sizes of Cabernet Gernischt berries into three major linkage groups (**Fig 3B)**. In general, Cluster 1 consisted of volatiles having higher levels in the 2015 season than that in the 2014 season. Cluster 2 consisted of the aroma compounds that had higher concentrations in the middle berries than in the small and large berries. Cluster 3 had the volatiles that were present at higher levels in 2014 than in 2015. Cluster 2, including 7 alcohols (5,8,11,13,14,17,21), 5 carbonyl compounds (23,29,30,31,36),4 benzenes (38,39,40,41), 3 C6/C9 compounds(48,49,52),1 norisoprenoids (66) and 1 terpenoids (73), were the major compounds differentiating the middle size of Cabernet Gernischt berries from the small and large berries. Taken collectively, we conclude that the volatile profiles of middle berries were different from those of small and large berries and that middle berries possessed the greatest abundance of aroma compounds. This was inconsistent with the result that the smaller berries contained a higher concentration of skin-located compounds (aromatic and phenolic compounds) due to a higher skin-to-pulp ratio [1,31]. This may be because small berries with a higher surface-to-volume ratio increased the respiration of berry compounds and the loss of berry volatiles [3]. In fact, until now there have been no consensus on the relationship between berry size and berry compositions. Some research indicates that the smaller berries had higher winegrape quality [1,8,32], while others are not [7,33,15,34]. Mark Matthews reported that the sources of variation in berry size are more important in determining grape composition than berry size *per se* [35]. For example, smaller berries of Cabernet Franc will commonly yield a richer must if berry size is reduced by environmental factors such as deficit irrigation [35]. By contrast, berries on well-watered Shiraz vines that are smaller for developmental reasons (and have fewer seeds) do not necessarily give rise to a richer must than their larger counterparts [7]. Our study was done in a vineyard without any treatment and concluded middle-sized Merlot and Cabernet Gernischt berries from natural variation had the greatest abundance of aroma compounds.

### OAVs

Not all of the compounds detected in the grape samples had a great impact on the overall aroma character of these fruits. Volatiles with OAVs above 1 are considered to be potent aroma contributors to grapes, although a compound with OAV <1 might also contribute to the grape aroma due to the additive or synergistic effects of similar compounds [36]. We found 20 odour-active volatiles with OAVs >1 (**Table 3**), among which 7 odour-active volatiles had OAVs higher than 20: (E)-2-nonenal, decanal, hexanal, (E)-2-hexenal, 2,6-nonadienal,(E,Z)-, (E)-β-damascenone and β-ionone. Among these, decanal (36), hexanal (47), (E)-2-hexenal (48), (E)-β-damascenone (65) and β-ionone (66) exhibited significantly higher OAVs in the middle sizes of Merlot berries. Similarly, (E)-2-hexenal (48) and (E)-β-damascenone (66) was found by ANOVA to have the markedly higher OAVs in the middle sizes of Cabernet Gernischt berries. (E)-β-damascenone and β-ionone likely conferred higher fruity and floral aromas, and decanal, hexanal and (E)-2-hexenal likely conferred higher fruity and fresh-green aromas to the middle-size berries [20,37]. Therefore, middle berries of both grapes may possess the greatest intensity of fresh-green, fruity and floral aromas. Nonanal had higher OAVs (>50) in the Merlot berries of the 2015 vintage likely due to seasonal and varietal differences; moreover, middle size berries contained the highest OAVs of nonanal. Benzeneacetaldehyde was not detected in either variety in 2014 but had the highest OAVs in the middle-size berries of 2015 vintage, with an OAV >19. Benzeneacetaldehyde has a floral and honey note [20] and played a more significant role in the floral aroma of grape berries in the middle-size berries of 2015 vintage than that of 2014 vintage. In summary, middle sizes of Merlot and Cabernet Gernischt berries had the greatest intensity of fresh-green, fruity and floral aromas. This may be because volatile compound biosynthetic genes showed differential expression among different sizes of grape berries. However, the related information on how berry sizes affect the genes and enzymes of various metabolic pathways leading to the diverse volatile compounds is limited. To the best of our knowledge, only Wong et al [8] reported that there were transcriptome changes involving aroma pathways between Merlot berries of different sizes. Further studies on the molecular mechanism underlying the regulation of berry size on the volatile compounds are under way.

**Table 3:** Odour activity values (OAVs) of the 20 most potent volatiles in differently sized *Vitis vinifera* L. cv. Merlot and Cabernet Gernischt berries in 2014 and 2015.

Odorants	Threshold (ug/L)	Aroma descriptor	NO^2^	Vintage 2014			Vintage 2015			NO	Vintage 2014			Vintage 2015		
				Merlot			Merlot				Cabernet Gernischt			Cabernet Gernischt		
				Large	Middle	Small	Large	Middle	Small		Large	Middle	Small	Large	Middle	Small
1-Octen-3-ol	1	mushroom	13	4.26±0.30a^3^	3.85±0.27a	3.88±0.27a	4.97±0.35a	4.77±0.34a	4.26±0.30a	13	3.98±0.28a	4.11±0.29a	4.18±0.30a	4.02±0.28a	4.48±0.32a	4.28±0.30a
Heptanal	3	Dry fish, solvent, smoky	22	0.61±0.04b	1.65±0.12a	1.31±0.09a	ND^4^	2.33±0.16a	ND	23	ND	1.51±0.11a	ND	ND	1.10±0.08a	ND
Octanal	0.7	Fruity, green, lemon	25	0.94±0.07b	2.14±0.15a	1.78±0.13a	1.61±0.11b	5.16±0.36a	ND	26	1.76±0.12b	3.35±0.24a	1.65±0.12b	1.99±0.14a	ND	1.64±0.12a
Nonanal	1	Citrusy, green	27	14.06±0.99c	35.37±2.50a	22.81±1.61b	59.24±4.19b	117.25±8.29a	127.82±9.04a	28	23.77±1.68a	15.3±1.08b	20.68±1.46a	43.42±3.07a	46.68±3.30a	56.61±4.00a
(E,E)-2,4-Hexadienal	10	Green, sweet, fruity	28	1.84±0.13a	2.35±0.17a	2.38±0.17a	6.59±0.47b	10.31±0.73a	6.67±0.47b	29	2.29±0.16a	2.79±0.20a	2.53±0.18a	2.62±0.19a	3.10±0.22a	2.79±0.20a
(E)-2-Octenal	3	Green, nut	29	0.72±0.05a	0.90±0.06a	0.92±0.07a	0.93±0.07b	1.37±0.10a	0.98±0.07b	30	1.04±0.07a	1.14±0.08a	1.04±0.07a	1.00±0.07ab	1.24±0.09a	0.90±0.06b
(E)-2-Nonenal	0.08	Cucumber, green	32	92.64±6.55a	64.92±4.59b	52.65±3.72b	68.17±4.82a	69.88±4.94a	ND	33	46.05±3.26a	47.43±3.35a	38.55±2.73a	53.59±3.79a	61.31±4.34a	50.88±3.60a
Benzeneacetaldehyde	4[38]^1^	Floral, rose, cherry-like	33	ND	ND	ND	ND	21.57±1.53a	ND	34	ND	ND	ND	19.61±1.39a	20.83±1.47a	ND
Decanal	0.1	Sweet, citrusy, green	36	150.07±10.61b	229.74±16.24a	192.58±13.62ab	252.76±17.87b	479.54±33.91a	231.76±16.39b	37	239.97±16.97a	189.36±13.39a	246.86±17.46a	373.58±26.42b	494.61±34.97a	513.76±36.33a
Hexanal	4.5	Grassy, green	47	492.76±34.84a	591.92±41.84a	580.84±41.08a	609.27±43.08b	963.62±68.14a	486.43±34.40b	47	479.64±33.92b	704.88±49.84a	675.52±47.76a	396.08±28.00c	571.72±40.40b	796.84±56.36a
(E)-2-Hexenal	17	Green, fresh, fruity	48	85.2±6.00b	145.92±10.32a	115.56±8.16ab	147.35±10.42b	302.35±21.38a	150.00±10.61b	49	64.60±4.60c	249.20±17.60a	139.10±9.80b	105.50±7.50b	277.70±19.60a	140.90±10.00b
(Z)-3-Hexenol	70[38]	Green	53	0.45±0.03b	0.72±0.05a	0.75±0.05a	1.61±0.11b	2.83±0.20a	2.40±0.17a	53	1.82±0.13a	1.67±0.12a	1.65±0.12a	1.62±0.11b	1.63±0.11b	2.75±0.19a
(E)-2-Hexenol	100[38]	Green	54	0.50±0.04c	1.26±0.09a	0.81±0.06b	2.11±0.15b	4.16±0.29a	4.53±0.32a	54	0.86±0.06a	0.55±0.04b	0.75±0.05a	1.55±0.11a	1.63±0.12a	2.01±0.14a
2,6-Nonadienal, (E,Z)-	0.02	Green, fresh cucumber	56	246.46±17.43a	251.54±17.79a	227.52±16.09a	202.85±14.34a	221.57±15.67a	172.73±12.21a	56	203.91±14.42a	208.29±14.73a	178.2±12.60a	248.69±17.59a	254.26±17.98a	233.26±16.49a
Ethyl hexanoate	5[38]	Fruity, anise	60	1.58±0.11a	1.40±0.10a	0.88±0.06b	0.69±0.05a	ND	0.74±0.05a	60	0.87±0.06a	0.88±0.06a	0.71±0.05a	0.50±0.04a	0.51±0.04a	ND
Hexyl acetate	2 [6]	Ripe fruit	62	0.42±0.03b	1.03±0.07a	ND	1.00±0.07b	2.43±0.17a	2.43±0.17a	62	0.53±0.04a	ND	ND	ND	0.50±0.04a	ND
6-Methyl-5-heptene-2-one	50[38]	Green, grassy,tomato-like	64	0.55±0.04b	0.87±0.06a	0.65±0.05b	1.13±0.08b	1.52±0.11a	1.60±0.11a	65	0.75±0.05a	0.64±0.04a	0.63±0.04a	0.97±0.07a	1.08±0.08a	1.26±0.09a
(E)-β-Damascenone	0.09	Sweet, flowers, stewed,apple	65	41.78±2.95c	260.49±18.42a	193.3±13.67b	219.49±15.52b	1216.24±86.00a	1167.52±82.56a	66	118.58±8.39b	212.51±15.03a	145.23±10.27b	167.54±11.85b	229.22±16.21a	12.72±0.90c
β-Ionone	0.07	Floral, violet	66	ND	354.17±25.04a	ND	ND	380.16±26.88a	ND	67	340.28±24.06a	330.06±23.34a	276.36±19.54a	277.34±19.61a	302.61±21.40a	337.3±23.85a
Geraniol	40	Floral, rose	72	0.58±0.04c	3.53±0.25a	2.64±0.19b	2.99±0.21b	16.20±1.15a	15.56±1.10a	73	1.65±0.12b	2.90±0.20a	2.01±0.14b	2.30±0.16b	3.12±0.22a	0.31±0.02c

Odour threshold, all the thresholds were obtained from ‘‘Odour & Flavour Detection Thresholds in Water (ug/L)” (http://www.leffingwell.com/odourthre.htm), except special instructions.

^1^ References.

^2^ The numbers correspond to those in **Fig 3.**

^3^ Tukey’s HSD test, different letters indicate significant differences at P < 0.05.

^4^ ND, not detected.

## Conclusion

It is extremely difficult to obtain uniform berry diameter and composition under field conditions, even when all vineyard management practices are properly executed. Berry classification based on size could minimize berry heterogeneity. This study aims to understand the relationship between the berry size and the aromatic compounds. A total of 76 and 79 volatile compounds were identified in *Vitis vinifera* L. cv. Merlot and Cabernet Gernischt, respectively. There were significant differences in volatile profiles among different sizes of *Vitis vinifera* L. cv. Merlot and Cabernet Gernischt berries in 2014 and 2015 seasons. Especially the volatile profiles of middle berries were dramatically different from those of large and small berries. Middle berries possessed the greatest abundance of aroma compounds, followed by small berries, with large berries having the lowest abundance. Sorting by berry size leaded to Merlot berries with a pronounced difference in C6/C9 compounds, norisoprenoids, terpenoids and Cabernet Gernischt berries with a pronounced difference in C6/C9 compounds and terpenoids. The OAV results showed that middle berries of both grape varieties may possess the greatest intensity of fresh-green, fruity and floral aromas, which ascribe to the high OAVs of decanal, hexanal, (E)-2-hexenal, (E)-β-damascenone and β-ionone in the middle sizes of Merlot berries and the high OAVs of (E)-2-hexenal and (E)-β-damascenone in the middle sizes of Cabernet Gernischt berries. Our findings will assist winemakers in conducting targeted berry sorting according to berry size to improve the aromatic quality of grapes.

## Supporting information

S1 Fig Average temperature and rainfall in July, August and September of 2014 and 2015 vintages. (DOCX)

S1 Table Quantitative ion, quantitative standards and calibration curves for quantification of volatile compounds. (DOCX)

S2 Table Concentrations (μg/L, mean ± SD) of volatile compounds in *Vitis vinifera* L. cv. Merlot berries in different size classes. (DOCX)

S3 Table Concentrations (μg/L, mean ± SD) of volatile compounds in *Vitis vinifera* L. cv. Cabernet Gernischt berries in different size classes. (DOCX)
