# Supplementary material for: Influence of natural variation in berry size on the volatile profiles of Vitis vinifera L. cv. Merlot and Cabernet Gernischt grapes
Source: PLoS One. 2018 Sep 19;13(9):e0201374. doi: 10.1371/journal.pone.0201374 (PMC6145503; doi:10.1371/journal.pone.0201374)
Supplement: S1 Fig — (DOCX) [file pone.0201374.s001.docx]

**S1 Fig.** Average temperature and rainfall in July, August and September of 2014 and 2015 vintages. YQY: Yuquanying (YQY) Farm of Ningxia Autonomous Region, China; The meteorological data (mean temperature and rainfall) were obtained from the China Meteorological Data Sharing Service System (http://cdc.cma.gov.cn/home.do).
