# Supplementary material for: Influence of natural variation in berry size on the volatile profiles of Vitis vinifera L. cv. Merlot and Cabernet Gernischt grapes
Source: PLoS One. 2018 Sep 19;13(9):e0201374. doi: 10.1371/journal.pone.0201374 (PMC6145503; doi:10.1371/journal.pone.0201374)
Supplement: S1 Table — (DOCX) [file pone.0201374.s002.docx]

| **S1 Table.** Quantitative ion, quantitative standards and calibration curves for quantification of volatile compounds | | | | | | | | |
| --- | --- | --- | --- | --- | --- | --- | --- | --- |
| NO | Compound | CAS | RI^1^ | Quantitative ion(m/z)^2^ | Quantitative standards | ID^3^ | Calibration curves | R^2^ |
| 1 | Acetic acid | 64197 | 1459.4 | 43 |  | C^4^ |  | - |
| 2 | Hexanoic acid | 142621 | 1859 | 60 | Hexanoic acid | A | y = 9412.52x＋40.68 | 0.979 |
| 3 | 2-Pentanol | 6032297 | 1123.5 | 45 | 1-Pentanol | B | y = 8912.33x-0.61 | 0.999 |
| 4 | 1-Butanol | 71363 | 1143.2 | 56 | 1-Butanol | A | y = 50581.72x-15.87 | 0.998 |
| 6 | Isopentanol | 123513 | 1200.1 | 55 | 1-Butanol | B | y = 47099.96x＋0.00 | 0.999 |
| 9 | 1-Pentanol | 71410 | 1242.6 | 42 | 1-Pentanol | A | y = 2049.71x＋4.91 | 0.95 |
| 10 | 3-Methyl-3-buten-1-ol | 763326 | 1244.8 | 86 | 3-Methyl-2-butenol | B | y = 48907.17x-4.52 | 0.997 |
| 11 | Isohexanol | 626891 | 1306.9 | 56 | Isohexanol | A | y = 3976.04x＋0.05 | 0.997 |
| 7 | 2-Heptanol | 543497 | 1311.5 | 45 | 2-Heptanol | A | y = 521.80x＋0.77 | 0.984 |
| 8 | 4-Methyl-2-hexanol | 2313613 | 1306.2 | 45 | 2-Heptanol | B | y = 521.80x＋0.77 | 0.984 |
| 12 | (Z)-2-Pentenol | 1576950 | 1316.7 | 57 | (E)-2-Hexenol | B | y = 7605.49x＋2.93 | 0.999 |
| 13 | 3-Methyl-2-butenol | 556821 | 1317.1 | 71 | 3-Methyl-2-butenol | A | y = 48500.33x-3.00 | 0.998 |
| 14 | 1-Octen-3-ol | 3391864 | 1447.7 | 57 | 1-Octen-3-ol | A | y = 218.64x＋3.15 | 0.997 |
| 15 | 1-Heptanol | 111706 | 1452.3 | 70 | 1-Heptanol | A | y = 1362.91x＋2.38 | 0.97 |
| 16 | 2-Ethyl-1-hexanol | 104767 | 1488 | 57 | 2-Ethyl-1-hexanol | A | y = 372.02x-0.30 | 0.976 |
| 17 | (S)-3-Ethyl-4-methylpentanol | NIST144071 | 1508.5 | 69 | 4-methyl-1-pentanol | B | y = 3745.02x-0.44 | 0.999 |
| 18 | 1-Octanol | 111875 | 1557.8 | 56 | 1-Octanol | A | y = 1297.17x＋1.73 | 0.959 |
| 19 | (E)-2-Octenol | 18409171 | 1618.9 | 57 | (E)-2-Octenol | A | y = 904.43x＋6.21 | 0.972 |
| 20 | 1-Nonanol | 143088 | 1662.3 | 56 | 2-Nonanol | B | y = 666.13x＋0.23 | 0.969 |
| 22 | Benzyl alcohol | 100516 | 1892.5 | 79 | Benzyl alcohol | A | y = 15127.68x＋141.11 | 0.956 |
| 23 | Phenylethyl Alcohol | 60128 | 1927.6 | 91 | Phenylethyl Alcohol | A | y = 14927.33x＋274.98 | 0.985 |
| 25 | Isobutyl ketone | 108838 | 1173.4 | 57 | Octanal | B | y = 472.87x＋12.81 | 0.994 |
| 26 | Heptanal | 111717 | 1187.9 | 70 | Octanal | B | y = 1705.88x＋1.83 | 0.96 |
| 27 | 4-Methyl-2-heptanone | 6137060 | 1213.9 | 43 | 2-Ethyl-1-hexanol | B | y = 372.02x-0.30 | 0.976 |
| 28 | 4,6-Dimethyl-2-heptanone | 19549805 | 1242 | 43 | 2-Ethyl-1-hexanol | B | y = 372.02x-0.30 | 0.976 |
| 29 | Octanal | 124130 | 1287.7 | 43 | Octanal | A | y = 1810.08x＋1.08 | 0.951 |
| 30 | (Z)-2-Heptenal | 57266861 | 1326.5 | 41 | Octanal | B | y = 919.71x＋1.43 | 0.967 |
| 31 | Nonanal | 124196 | 1394.8 | 57 | Nonanal | A | y = 760.00x-0.42 | 0.973 |
| 32 | (E,E)-2,4-Hexadienal | 142836 | 1408.3 | 81 | (E)-2-Hexenal | B | y = 8238.82x＋11.36 | 0.996 |
| 33 | (E)-2-Octenal | 2548870 | 1433.7 | 41 | (E)-2-Nonenal | B | y = 1766.26x＋2.11 | 0.983 |
| 34 | (E,E)-2,4-Heptadienal | 4313035 | 1500.8 | 81 | (E)-2-Nonenal | B | y = 754.07x＋7.37 | 0.955 |
| 35 | Benzaldehyde | 100527 | 1536.4 | 77 | Benzaldehyde | A | y = 6053.04x＋6.01 | 0.979 |
| 36 | (E)-2-Nonenal | 18829566 | 1542.1 | 43 | (E)-2-Nonenal | A | y = 4338.17x＋1.33 | 0.977 |
| 37 | Benzeneacetaldehyde | 122781 | 1659.1 | 91 | Benzeneacetaldehyde | A | y = 6327.32x＋72.26 | 0.968 |
| 38 | Acetophenone | 98862 | 1666.2 | 105 | Benzaldehyde | B | y = 11269.92x-1.20 | 0.999 |
| 39 | 3,4-Dimethylbenzaldehyde | 5973717 | 1772.5 | 133 | Benzyl alcohol | B | y = 15127.68x＋141.11 | 0.956 |
| 40 | Decanal | 112312 | 1501.9 | 43 | Decanal | A | y = 6998.38x-0.85 | 0.969 |
| 41 | Toluene | 108883 | 1047.4 | 91 | Styrene | B | y = 1268.23x＋0.96 | 0.966 |
| 42 | p-Xylene | 106423 | 1146.1 | 91 | p-Cymene | B | y = 860.13x＋1.07 | 0.966 |
| 43 | 1,3-Dimethyl benzene | 108383 | 1157.3 | 91 | Benzyl alcohol | B | y = 15127.68x＋141.11 | 0.956 |
| 44 | o-Xylene | 95476 | 1195 | 91 | p-Cymene | B | y = 860.13x＋1.07 | 0.966 |
| 45 | Styrene | 100425 | 1260.1 | 104 | Styrene | A | y = 736.18x＋1.69 | 0.973 |
| 46 | p-Cymene | 99876 | 1271.1 | 119 | p-Cymene | A | y = 229.49x＋3.19 | 0.972 |
| 47 | o-Cymene | 527844 | 1271.7 | 119 | p-Cymene | B | y = 229.49x＋3.19 | 0.972 |
| 48 | Naphthalene | 91203 | 1761 | 128 | Naphthalene | A | y = 762.23x-0.19 | 0.979 |
| 49 | 2-Methyl-naphthalene | 91576 | 1842.8 | 142 | Naphthalene | B | y = 762.23x-0.19 | 0.979 |
| 51 | 2-Nonanone | 821556 | 1003.2 | 43 | 2-Nonanol | B | y = 1010.45x＋0.10 | 0.998 |
| 52 | Hexanal | 66251 | 1098.8 | 44 | Hexanal | A | y = 3922.72x-208.67 | 0.999 |
| 53 | 2-Hexanol | 626937 | 1211.7 | 45 | 1-Hexanol | B | y = 3436.65x＋0.20 | 0.994 |
| 54 | (E)-2-Hexenal | 6728263 | 1204 | 41 | (E)-2-Hexenal | A | y = 5828.38x-380.83 | 0.993 |
| 55 | 3-Hexen-1-ol, acetate, (Z)- | 3681718 | 1307.7 | 43 | Ethyl hexanoate | B | y = 1991.68x-0.14 | 0.984 |
| 56 | (E)-3-Hexenyl acetate | 3681821 | 1315.8 | 43 | Ethyl hexanoate | B | y = 1991.68x-0.14 | 0.984 |
| 57 | 1-Hexanol | 111273 | 1347.1 | 56 | 1-Hexanol | A | y = 1636.43x＋0.00 | 0.999 |
| 58 | (E)-3-Hexenol | 928972 | 1359.5 | 41 | (E)-3-Hexenol | A | y = 446.86x＋4.81 | 0.994 |
| 59 | (Z)-3-Hexenol | 928961 | 1381 | 67 | (Z)-3-Hexenol | A | y = 7898.50x＋35.81 | 0.99 |
| 60 | (E)-2-Hexenol | 928950 | 1403.4 | 57 | (E)-2-Hexenol | A | y = 2685.83x＋0.00 | 0.999 |
| 61 | (Z)-2-Hexenol | 928949 | 1413.1 | 57 | (Z)-2-Hexenol | A | y = 6137.53x-2.71 | 0.997 |
| 62 | 2,6-Nonadienal, (E,Z)- | 17587336 | 1594.6 | 41 | (E)-2-Nonenal | B | y = 1766.26x＋2.11 | 0.983 |
| 63 | Ethyl Acetate | 141786 | 789.1 | 43 | Ethyl Acetate | A | y = 1896.30x＋0.00 | 0.999 |
| 64 | n-Butyl acetate | 123864 | 1076.6 | 43 | Ethyl butanoate | B | y = 1271.59x＋0.49 | 0.999 |
| 65 | Butyl 2-propenoate | 141322 | 1178.7 | 55 | Ethyl butanoate | B | y = 1353.00x＋0.17 | 0.998 |
| 66 | Ethyl hexanoate | 123660 | 1231.3 | 88 | Ethyl hexanoate | A | y = 1085.22x＋1.29 | 0.964 |
| 67 | Ethyl isohexanoate | 123660 | 1231.3 | 88 | Ethyl hexanoate | B | y = 1085.22x＋1.29 | 0.964 |
| 68 | Hexyl acetate | 142927 | 1270.5 | 43 | Hexyl acetate | A | y = 1612.24x-0.03 | 0.986 |
| 69 | 2-Hexenoic acid, methyl ester, (E)- | 13894638 | 1288 | 55 | Ethyl hexanoate | B | y = 1991.68x-0.14 | 0.984 |
| 70 | Methyl salicylate | 119368 | 1795 | 120 | Methyl salicylate | A | y = 7146.00x＋0.36 | 0.995 |
| 71 | 6-Methyl-5-heptene-2-one | 110930 | 1337.8 | 43 | 6-Methyl-5-heptene-2-one | A | y = 941.30x＋1.97 | 0.966 |
| 73 | (E)-β-Damascenone | 23726934 | 1834.5 | 69 | (E)-β-damascenone | A | y = 12146.60x＋0.00 | 0.999 |
| 81 | β-Ionone | 14901076 | 1881.6 | 177 | β-Damascenone | B | y = 20064.90x＋9.46 | 0.982 |
| 75 | Geranylacetone | 3796701 | 1864.8 | 43 | Geranylacetone | A | y = 2763.22x＋0.35 | 0.983 |
| 76 | Camphor | 76222 | 1526 | 95 | 6-Methyl-5-hepten-2-ol | B | y = 3069.68x＋0.12 | 0.989 |
| 77 | Linalool | 78706 | 1548.3 | 71 | Linalool | A | y = 512.09x＋1.37 | 0.957 |
| 79 | β-Citronellol | 1117619 | 1717.5 | 69 | Terpineol | B | y = 2303.39x＋0.21 | 0.958 |
| 80 | Calacorene | 21391991 | 1933.8 | 157 | Nerolidol | B | y = 1131.02x＋2.78 | 0.989 |
| 74 | Geraniol | 106241 | 1853.8 | 69 | Geraniol | A | y = 7950.19x＋3.27 | 0.975 |
| 82 | Cadalene | **483783** | 2256 | 183 | - | C | - | - |
| 83 | Phenol, 2,5-bis(1,1-dimethylethyl)- | 5875456 | 2214.1 | 191 | - | C | - | - |
| 84 | 2-Ethylfuran | 3208160 | 943 | 81 | Octanal | B | y = 799.88x＋6.26 | 0.962 |
| 85 | Diacetyl | 431038 | 967.4 | 43 | 1-Butanol | B | y = 56547.22x-41.94 | 0.994 |
| 87 | Eucalyptol | 470826 | 1211.3 | 43 | Terpinolene | B | y = 1336.94x＋4.75 | 0.971 |
| 88 | Trans-2-(2-Pentenyl)furan | 70424145 | 1296.8 | 107 | Benzeneacetaldehyde | B | y = 6327.32x＋72.26 | 0.968 |

| ^1^ Retention indices were caculated on HP-INNOWAX column. |
| --- |
| ^2^ Quantitative ion for peak area evaluation of volatile compounds. |
| ^3^ Identification of volatile compounds: A, identified, mass spectrum and RI agreed with standards; B and C, tentatively identified, mass spectrum and RI agreed with NIST 11 MS database and literature data. |
| ^4^ The concentration of these compounds expressed as relative areas (to 4-methyl-2-pentanol). |
