# Supplementary material for: Influence of natural variation in berry size on the volatile profiles of Vitis vinifera L. cv. Merlot and Cabernet Gernischt grapes
Source: PLoS One. 2018 Sep 19;13(9):e0201374. doi: 10.1371/journal.pone.0201374 (PMC6145503; doi:10.1371/journal.pone.0201374)
Supplement: S2 Table — (DOCX) [file pone.0201374.s003.docx]

**S2 Table.** Concentrations (μg/L, mean ± SD) of volatile compounds in *Vitis vinifera* L. cv. Merlot berries in different size classes

|  |  |  | 2014 Vintage | | | 2015 Vintage | | |
| --- | --- | --- | --- | --- | --- | --- | --- | --- |
| NO | Compounds | ID^1^ | Large berries | Middle berries | Small berries | Large berries | Middle berries | Small berries |
|  | **Total acids** |  |  |  |  |  |  |  |
| 1 | Acetic acid | C^2^ | <0.005 | 0.01±0.00a^3^ | 0.01±0.00a | 0.01±0.00b | 0.02±0.00a | 0.02±0.00a |
| 2 | Hexanoic acid | A | 146.95±10.39a | 166.93±11.80a | 188.39±13.32a | 191.02±13.51a | 248.91±17.60a | 212.95±15.06a |
|  | Subtotal |  | 146.96±10.39b | 166.93±11.80ab | 188.4±13.32a | 191.03±13.51b | 248.92±17.60a | 212.97±15.06ab |
|  | Subtotal % |  | 2.71 | 2.13 | 2.90 | 2.37 | 1.66 | 2.19 |
|  | **Total alcohols** |  |  |  |  |  |  |  |
| 3 | 2-Pentanol | B | ND^4^ | ND | ND | ND | 39.73±2.81a | ND |
| 4 | 1-Butanol | A | ND | ND | ND | ND | ND | 27.82±1.97a |
| 5 | Isopentanol | B | ND | 164.56±11.64a | ND | ND | 634.01±44.83a | 454.60±32.14b |
| 6 | 1-Pentanol | A | 7.16±0.51a | 6.31±0.45a | 6.21±0.44a | 6.90±0.49a | ND | 7.16±0.51a |
| 7 | 3-Methyl-3-buten-1-ol | B | 114.40±8.09a | 79.72±5.64b | 67.64±4.78b | 75.63±5.35b | ND | 101.95±7.21a |
| 8 | Isohexanol | A | 0.14±0.01a | ND | ND | 0.32±0.02a | 0.17±0.01b | ND |
| 9 | 2-Heptanol | A | 2.45±0.17a | 2.16±0.15a | 2.06±0.15a | 5.61±0.40a | 5.13±0.36a | 4.54±0.32a |
| 10 | 4-Methyl-2-hexanol | B | 2.25±0.19a | 2.06±0.15a | 1.87±0.19a | 5.81±0.57a | 5.23±0.42a | 4.43±0.22a |
| 11 | (Z)-2-Pentenol | B | 5.90±0.42b | 11.59±0.82a | 10.00±0.71a | 5.47±0.39b | 10.74±0.76a | 9.30±0.66a |
| 12 | 3-Methyl-2-butenol | A | ND | ND | ND | 13.29±0.94a | 12.57±0.89a | 8.91±0.63b |
| 13 | 1-Octen-3-ol | A | 4.26±0.30a | 3.85±0.27a | 3.88±0.27a | 4.97±0.35a | 4.77±0.34a | 4.26±0.30a |
| 14 | 1-Heptanol | A | 2.10±0.15b | 3.09±0.22a | 2.54±0.18ab | 2.89±0.20b | 4.34±0.31a | 3.39±0.24b |
| 15 | 2-Ethyl-1-hexanol | A | 2.79±0.20b | 4.45±0.31a | 3.64±0.26ab | 4.75±0.34b | 9.34±0.66a | 4.43±0.31b |
| 16 | (S)-3-Ethyl-4-methylpentanol | B | ND | ND | ND | 3.05±0.22c | 11.91±0.84a | 9.44±0.67b |
| 17 | 1-Octanol | A | 1.83±0.13b | 2.53±0.18a | 2.18±0.15ab | 3.07±0.22b | 4.87±0.34a | 3.04±0.21b |
| 18 | (E)-2-Octenol | A | ND | ND | ND | ND | 6.91±0.49a | ND |
| 19 | Benzyl alcohol | A | 166.78±11.79a | 157.13±11.11a | 163.08±11.53a | 187.53±13.26a | 184.36±13.04a | 175.69±12.42a |
| 20 | Phenylethyl Alcohol | A | 263.40±18.63a | 285.59±20.19a | 284.29±20.10a | 297.67±21.05a | 328.71±23.24a | 315.31±22.30a |
|  | Subtotal |  | 573.65±40.56a | 723.14±51.13a | 547.59±38.72a | 616.75±43.61b | 1262.69±89.29a | 1134.37±80.21a |
|  | Subtotal % |  | 10.57 | 7.11 | 8.42 | 7.66 | 8.41 | 11.64 |
|  | **Total carbonyl compounds** | | |  |  |  |  |  |
| 21 | Isobutyl ketone | B | 72.36±5.12a | 90.78±6.42a | 72.07±5.10a | 51.67±3.65a | 61.03±4.32a | 55.27±3.91a |
| 22 | Heptanal | B | 1.84±0.13b | 4.94±0.35a | 3.93±0.28a | ND | 6.99±0.49a | ND |
| 23 | 4-Methyl-2-heptanone | B | ND | ND | ND | ND | 1.06±0.08a | ND |
| 24 | 4,6-Dimethyl-2-heptanone | B | 1.00±0.07ab | 1.13±0.08a | 0.79±0.06b | 1.66±0.12a | 1.59±0.11a | 1.60±0.11a |
| 25 | Octanal | A | 0.66±0.05b | 1.50±0.11a | 1.24±0.09a | 1.13±0.08b | 3.61±0.26a | ND |
| 26 | (Z)-2-Heptenal | B | ND | 1.72±0.12a | ND | ND | 3.67±0.26a | 3.09±0.22a |
| 27 | Nonanal | A | 14.06±0.99c | 35.37±2.50a | 22.81±1.61b | 59.24±4.19b | 117.25±8.29a | 127.82±9.04a |
| 28 | (E,E)-2,4-Hexadienal | B | 18.35±1.30a | 23.54±1.66a | 23.78±1.68a | 65.88±4.66b | 103.13±7.29a | 66.70±4.72b |
| 29 | (E)-2-Octenal | B | 2.16±0.15a | 2.71±0.19a | 2.77±0.20a | 2.79±0.20b | 4.12±0.29a | 2.94±0.21b |
| 30 | (E,E)-2,4-Heptadienal | B | 8.01±0.57a | 8.35±0.59a | 8.58±0.61a | 8.11±0.57a | 8.95±0.63a | 8.19±0.58a |
| 31 | Benzaldehyde | A | 7.77±0.55b | 16.27±1.15a | 17.48±1.24a | 21.47±1.52b | 61.56±4.35a | 31.65±2.24b |
| 32 | (E)-2-Nonenal | A | 7.41±0.52a | 5.19±0.37b | 4.21±0.30b | 5.45±0.39a | 5.59±0.40a | ND |
| 33 | Benzeneacetaldehyde | A | ND | ND | ND | ND | 86.28±6.10a | ND |
| 34 | Acetophenone | B | ND | ND | ND | ND | 29.61±2.09a | ND |
| 35 | 3,4-Dimethylbenzaldehyde | B | 125.16±8.85a | 150.41±10.64a | 147.34±10.42a | 148.45±10.50a | 173.65±12.28a | 179.51±12.69a |
| 36 | Decanal | A | 15.01±1.06b | 22.97±1.62a | 19.26±1.36ab | 25.28±1.79b | 47.95±3.39a | 23.18±1.64b |
|  | Subtotal |  | 273.79±19.36b | 364.88±25.80a | 324.25±22.93ab | 391.13±27.66b | 716.06±50.63a | 499.95±35.35b |
|  | Subtotal % |  | 5.04 | 4.65 | 4.99 | 4.86 | 4.77 | 5.13 |
|  | **Total benzenes** |  |  |  |  |  |  |  |
| 37 | Toluene | B | 22.77±1.61b | 44.83±3.17a | 22.93±1.62b | 14.44±1.02b | 30.24±2.14a | 12.75±0.90b |
| 38 | p-Xylene | B | 3.18±0.22b | 7.41±0.52a | 1.59±0.11c | 4.05±0.29b | 7.67±0.54a | 3.81±0.27b |
| 39 | 1,3-Dimethyl benzene | B | 211.51±14.96b | 355.52±25.14a | 152.73±10.80b | 255.92±18.10b | 375.09±26.52a | 239.87±16.96b |
| 40 | o-Xylene | B | 5.47±0.39b | 7.80±0.55a | 4.66±0.33b | 3.94±0.28b | 5.64±0.40a | 3.34±0.24b |
| 41 | Styrene | A | 1.65±0.12c | 5.67±0.40a | 4.05±0.29b | 6.35±0.45b | 30.75±2.17a | 6.65±0.47b |
| 42 | p-Cymene | A | ND | ND | ND | 3.30±0.23a | ND | ND |
| 43 | o-Cymene | B | ND | ND | ND | ND | 3.55±0.25a | 3.60±0.25a |
| 44 | Naphthalene | A | 0.59±0.04c | 1.64±0.12a | 0.97±0.07b | 4.70±0.33b | 16.71±1.18a | 3.95±0.28b |
| 45 | 2-Methyl-naphthalene | B | ND | ND | ND | 0.53±0.04b | 3.67±0.26a | 0.59±0.04b |
|  | Subtotal |  | 245.16±17.34b | 422.87±29.90a | 186.92±13.22b | 293.23±20.73b | 473.33±33.47a | 274.55±19.41b |
|  | Subtotal % |  | 4.52 | 5.38 | 2.87 | 3.64 | 3.15 | 2.82 |
|  | **Total C6/C9 Compounds** | |  |  |  |  |  |  |
| 46 | 2-Nonanone | B | 0.69±0.05a | ND | 0.69±0.05 | ND | ND | ND |
| 47 | Hexanal | A | 2217.37±156.79a | 2663.56±188.34a | 2613.70±184.82a | 2741.73±193.87b | 4336.29±306.62a | 2188.93±154.78b |
| 48 | (E)-2-Hexenal | A | 1448.09±102.40b | 2481.18±175.45a | 1964.19±138.89ab | 2504.98±177.13b | 5139.88±363.44a | 2549.98±180.31b |
| 49 | 3-Hexen-1-ol, acetate, (Z)- | B | ND | ND | ND | 4.53±0.32c | 20.04±1.42a | 14.74±1.04b |
| 50 | (E)-3-Hexenyl acetate | B | ND | ND | ND | 4.43±0.32c | 22.08±1.52a | 16.79±1.24b |
| 51 | 1-Hexanol | A | 99.41±7.03b | 161.19±11.40a | 117.88±8.34b | 208.52±14.74b | 284.90±20.15a | 300.43±21.24a |
| 52 | (E)-3-Hexenol | A | 5.09±0.36a | 5.63±0.40a | 5.29±0.37a | 5.42±0.38a | 5.71±0.40a | 5.93±0.42a |
| 53 | (Z)-3-Hexenol | A | 31.73±2.24b | 50.45±3.57a | 52.78±3.73a | 112.54±7.96b | 198.43±14.03a | 167.75±11.86a |
| 54 | (E)-2-Hexenol | A | 50.40±3.56c | 126.49±8.94a | 81.46±5.76b | 210.68±14.90b | 415.87±29.41a | 453.35±32.06a |
| 55 | (Z)-2-Hexenol | A | 114.50±8.10c | 288.53±20.40a | 184.89±13.07b | 478.97±33.87b | 947.67±67.01a | 1032.77±73.03a |
| 56 | 2,6-Nonadienal, (E,Z)- | B | 4.93±0.35a | 5.03±0.36a | 4.55±0.32a | 4.06±0.29a | 4.43±0.31a | 3.45±0.24a |
|  | Subtotal |  | 3972.2±280.88b | 5782.06±408.85a | 5025.43±355.35ab | 6275.97±443.78b | 11373.31±804.21a | 6732.07±476.03b |
|  | Subtotal % |  | 73.18 | 73.63 | 77.28 | 77.95 | 75.72 | 69.11 |
|  | **Total esters** |  |  |  |  |  |  |  |
| 57 | Ethyl Acetate | A | 120.74±8.54a | 111.52±7.89a | 47.07±3.33b | 49.38±3.49a | 30.01±2.12b | 34.86±2.46b |
| 58 | n-Butyl acetate | B | ND | ND | 1.34±0.09a | ND | ND | ND |
| 59 | Butyl 2-propenoate | B | 12.64±0.89a | 12.38±0.88a | ND | 0.17±0.01a | ND | ND |
| 60 | Ethyl hexanoate | A | 7.92±0.56a | 6.98±0.49a | 4.39±0.31b | 3.47±0.25a | ND | 3.69±0.26a |
| 61 | Ethyl isohexanoate | B | 6.51±0.46a | 7.01±0.50a | 4.39±0.31b | 3.47±0.25a | 2.38±0.17b | 3.69±0.26a |
| 62 | Hexyl acetate | A | 0.84±0.06b | 2.06±0.15a | ND | 2.00±0.14b | 4.87±0.34a | 4.86±0.34a |
| 63 | 2-Hexenoic acid, methyl ester, (E)- | B | ND | ND | ND | 2.23±0.16b | 3.19±0.23a | ND |
|  | Subtotal |  | 148.66±10.51a | 139.94±9.93a | 57.19±4.04b | 60.74±4.29a | 40.45±2.86b | 47.10±3.33b |
|  | Subtotal % |  | 2.74 | 1.78 | 0.88 | 0.75 | 0.27 | 0.48 |
|  | **Total norisoprenoids** | |  |  |  |  |  |  |
| 64 | 6-Methyl-5-heptene-2-one | A | 27.57±1.95b | 43.68±3.09a | 32.72±2.31b | 56.27±3.98b | 75.83±5.36a | 80.10±5.66a |
| 65 | (E)-β-Damascenone | A | 3.76±0.27c | 23.44±1.66a | 17.4±1.23b | 19.75±1.40b | 109.46±7.74a | 105.08±7.43a |
| 66 | β-Ionone | B | ND | 24.79±1.75a | ND | ND | 26.61±1.88a | ND |
| 67 | Geranylacetone | A | 5.22±0.37a | 5.68±0.40a | 6.87±0.49a | 7.42±0.52a | 8.49±0.60a | 9.35±0.66a |
|  | Subtotal |  | 36.55±2.58c | 97.60±6.90a | 56.99±4.03b | 83.44±5.90b | 220.40±15.58a | 194.52±13.75a |
|  | Subtotal % |  | 0.67 | 1.24 | 0.88 | 1.04 | 1.47 | 2.00 |
|  | **Total terpenoids** |  |  |  |  |  |  |  |
| 68 | Camphor | C | 0.02±0.00a | 0.02±0.00a | 0.02±0.00a | 0.02±0.00b | 0.03±0.00a | 0.02±0.00b |
| 69 | Linalool | A | ND | ND | ND | ND | 2.09±0.15a | 1.74±0.12ab |
| 70 | β-Citronellol | B | 0.04±0.00b | 1.37±0.10a | 1.08±0.08a | ND | 6.14±0.43a | 5.70±0.40a |
| 71 | Calacorene | B | ND | 3.56±0.25a | ND | ND | 3.72±0.26a | 3.65±0.26a |
| 72 | Geraniol | A | 23.27±1.65c | 141.37±10.00a | 105.75±7.48b | 119.63±8.46b | 648.10±45.83a | 622.24±44.00a |
|  | Subtotal |  | 23.34±1.65c | 146.33±10.35a | 106.86±7.56b | 119.65±8.46b | 660.09±46.68a | 633.35±44.78a |
|  | Subtotal % |  | 0.43 | 1.86 | 1.64 | 1.49 | 4.39 | 6.50 |
|  | **Total volatile phenols** | |  |  |  |  |  |  |
| 73 | Phenol, 2,5-bis(1,1-dimethylethyl)- | C | 1.44±0.10a | 1.76±0.12a | 0.38±0.03b | 2.81±0.20a | 1.80±0.13b | 2.36±0.17ab |
|  | Subtotal |  | 1.44±0.10a | 1.76±0.12a | 0.38±0.03b | 2.81±0.2a | 1.80±0.13b | 2.36±0.17ab |
|  | Subtotal % |  | 0.03 | 0.02 | 0.01 | 0.03 | 0.01 | 0.02 |
|  | **Others** |  |  |  |  |  |  |  |
| 74 | 2-Ethylfuran | B | 6.46±0.46a | 7.39±0.52a | 7.50±0.53a | 9.94±0.7a | 12.53±0.89a | 10.38±0.73a |
| 75 | Diacetyl | B | ND | ND | 1.09±0.08a | ND | ND | ND |
| 76 | Eucalyptol | B | ND | ND | ND | 6.15±0.44b | 10.80±0.76a | ND |
|  | Subtotal |  | 6.46±0.46b | 7.39±0.52a | 8.59±0.61a | 16.10±1.14b | 23.32±1.65a | 10.38±0.73c |
|  | Subtotal % |  | 0.12 | 0.09 | 0.13 | 0.20 | 0.16 | 0.11 |
|  | Sum |  | 5428.20±383.83b | 7852.91±555.32a | 6502.6±459.80ab | 8050.84±569.28 b | 15020.39±1062.10a | 9741.63±688.84b |

^1^ Reliability of the identification proposal: A, identified, mass spectrum and RI agreed with standards; B and C, tentatively identified, mass spectrum and RI agreed with NIST 11 MS database and literature data.

^2^ The concentration of these compounds expressed as relative areas (to 4-methyl-2-pentanol).

^3^ Tukey’s HSD test, different letters indicate significant differences at P < 0.05.

^4^ ND: not detected.
