# Supplementary material for: Influence of natural variation in berry size on the volatile profiles of Vitis vinifera L. cv. Merlot and Cabernet Gernischt grapes
Source: PLoS One. 2018 Sep 19;13(9):e0201374. doi: 10.1371/journal.pone.0201374 (PMC6145503; doi:10.1371/journal.pone.0201374)
Supplement: S3 Table — (DOCX) [file pone.0201374.s004.docx]

**S3 Table.** Concentrations (μg/L, mean ± SD) of volatile compounds in *Vitis vinifera* L. cv. Cabernet Gernischt berries in different size classes

|  |  |  | 2014 Vintage | | | 2015 Vintage | | |
| --- | --- | --- | --- | --- | --- | --- | --- | --- |
| NO | Compounds | ID^1^ | Large berries | Middle berries | Small berries | Large berries | Middle berries | Small berries |
|  | **Total acids** | |  |  |  |  |  |  |
| 1 | Acetic acid | C^2^ | <0.005 | <0.005 | <0.005 | 0.01±0.00b^3^ | 0.01±0.00a | 0.01±0.00b |
| 2 | Hexanoic acid | A | 56.13±3.97a | 58.72±4.15a | 62.73±4.44a | 85.92±6.08a | 94.04±6.65a | 94.11±6.65a |
|  | Subtotal |  | 56.13±3.97a | 58.73±4.15a | 62.73±4.44a | 85.92±6.08a | 94.05±6.65a | 94.12±6.66a |
|  | Subtotal % |  | 1.66 | 2.19 | 1.01 | 0.59 | 0.82 | 1.33 |
|  | **Total alcohols** | |  |  |  |  |  |  |
| 3 | 2-Pentanol | B | ND^4^ | ND | ND | 14.86±1.05b | 56.14±3.97a | ND |
| 4 | 1-Butanol | A | 68.13±4.82a | ND | ND | 60.53±4.28ab | 68.94±4.87a | 50.92±3.60b |
| 5 | Isopentanol | B | 231.33±16.36a | 264.82±18.73a | 203.09±14.36a | 240.88±17.03a | 255.6±18.07a | 162.73±11.51b |
| 6 | 1-Pentanol | A | 6.72±0.48a | 6.81±0.48a | 6.74±0.48a | ND | ND | 7.00±0.50a |
| 7 | 3-Methyl-3-buten-1-ol | B | 97.53±6.90a | 110.74±7.83a | 93.67±6.62a | 100.96±7.14a | 79.89±5.65a | 93.96±6.64a |
| 8 | Isohexanol | A | 0.12±0.01b | 0.12±0.01b | 0.37±0.03a | 0.18±0.01b | 0.64±0.05a | ND |
| 9 | 2-Heptanol | A | 2.51±0.18b | 5.12±0.36a | 2.31±0.16b | 2.33±0.16a | 3.04±0.22a | 2.76±0.20a |
| 10 | 4-Methyl-2-hexanol | B | 2.51±0.18b | 5.12±0.36a | 2.31±0.16b | 2.33±0.16a | 3.04±0.22a | 2.76±0.20a |
| 11 | (Z)-2-Pentenol | B | 15.55±1.10a | 13.93±0.99a | 16.91±1.20a | 13.5±0.95a | 16.63±1.18a | 14.37±1.02a |
| 12 | 3-Methyl-2-butenol | A | ND | ND | ND | 0.67±0.05b | 5.3±0.37a | ND |
| 13 | 1-Octen-3-ol | A | 3.98±0.28a | 4.11±0.29a | 4.18±0.30a | 4.02±0.28a | 4.48±0.32a | 4.28±0.30a |
| 14 | 1-Heptanol | A | 2.73±0.19a | 3.34±0.24a | 3.10±0.22a | 2.67±0.19a | 3.26±0.23a | 2.78±0.20a |
| 15 | 2-Ethyl-1-hexanol | A | 4.80±0.34a | 3.61±0.26a | 4.73±0.33a | 7.15±0.51b | 9.41±0.67ab | 10.44±0.74a |
| 16 | (S)-3-Ethyl-4-methylpentanol | B | 9.85±0.70a | 10.32±0.73a | 11.85±0.84a | 34.41±2.43b | 52.56±3.72a | 35.40±2.50b |
| 17 | 1-Octanol | A | 2.46±0.17a | 2.62±0.19a | 2.44±0.17a | 2.35±0.17a | 2.87±0.20a | ND |
| 18 | (E)-2-Octenol | A | ND | ND | ND | ND | 6.37±0.45a | ND |
| 19 | 1-Nonanol | B | 0.62±0.04a | ND | 0.67±0.05a | ND | ND | ND |
| 20 | Benzyl alcohol | A | 156.17±11.04a | 158.74±11.22a | 157.47±11.13a | 175.95±12.44a | 176.42±12.47a | 164.95±11.66a |
| 21 | Phenylethyl Alcohol | A | 304.58±21.54a | 307.22±21.72a | 302.55±21.39a | 311.37±22.02a | 307.73±21.76a | 306.32±21.66a |
|  | Subtotal |  | 909.59±64.32a | 896.62±63.40a | 812.41±57.45a | 974.15±68.88a | 1052.31±74.41a | 858.67±60.72a |
|  | Subtotal % |  | 8.41 | 11.64 | 16.37 | 9.06 | 10.68 | 15.13 |
|  | **Total carbonyl compounds** | | |  |  |  |  |  |
| 22 | Isobutyl ketone | B | 87.31±6.17a | 85.38±6.04a | 81.55±5.77a | 40.85±2.89b | 62.38±4.41a | 42.99±3.04b |
| 23 | Heptanal | B | ND | 4.54±0.32a | ND | ND | 3.29±0.23a | ND |
| 24 | 4-Methyl-2-heptanone | B | 0.37±0.03b | 0.66±0.05a | 0.32±0.02b | 0.50±0.04a | 0.37±0.03a | 0.50±0.04a |
| 25 | 4,6-Dimethyl-2-heptanone | B | 1.15±0.08a | 1.08±0.08a | 0.99±0.07a | 1.19±0.08a | 1.21±0.09a | 1.10±0.08a |
| 26 | Octanal | A | 1.23±0.09b | 2.35±0.17a | 1.15±0.08b | 1.40±0.10a | ND | 1.15±0.08a |
| 27 | (Z)-2-Heptenal | B | ND | 1.88±0.13a | ND | ND | ND | 1.97±0.14a |
| 28 | Nonanal | A | 23.77±1.68a | 15.3±1.08b | 20.68±1.46a | 43.42±3.07a | 46.68±3.30a | 56.61±4.00a |
| 29 | (E,E)-2,4-Hexadienal | B | 22.85±1.62a | 27.86±1.97a | 25.33±1.79a | 26.2±1.85a | 30.99±2.19a | 27.9±1.97a |
| 30 | (E)-2-Octenal | B | 3.11±0.22a | 3.41±0.24a | 3.12±0.22a | 2.99±0.21ab | 3.71±0.26a | 2.70±0.19b |
| 31 | (E,E)-2,4-Heptadienal | B | 8.26±0.58a | 8.47±0.60a | 8.39±0.59a | 7.92±0.56a | 8.32±0.59a | 7.95±0.56a |
| 32 | Benzaldehyde | A | 12.85±0.91a | 15.06±1.07a | 14.51±1.03a | 21.03±1.49b | 36.65±2.59a | 18.58±1.31b |
| 33 | (E)-2-Nonenal | A | 3.68±0.26a | 3.79±0.27a | 3.08±0.22a | 4.29±0.30a | 4.90±0.35a | 4.07±0.29a |
| 34 | Benzeneacetaldehyde | A | ND | ND | ND | 78.44±5.55a | 83.34±5.89a | ND |
| 35 | Acetophenone | B | ND | ND | ND | 4.33±0.31b | 12.24±0.87a | ND |
| 36 | 3,4-Dimethylbenzaldehyde | B | ND | ND | 147.81±10.45a | ND | 157.01±11.10a | ND |
| 37 | Decanal | A | 24.00±1.70a | 18.94±1.34a | 24.69±1.75a | 37.36±2.64b | 49.46±3.50a | 51.38±3.63a |
|  | Subtotal |  | 188.61±13.34b | 188.74±13.35b | 331.61±23.45a | 269.92±19.09b | 500.54±35.39a | 216.91±15.34b |
|  | Subtotal % |  | 4.77 | 5.13 | 3.39 | 1.91 | 4.36 | 4.19 |
|  | **Total benzenes** | |  |  |  |  |  |  |
| 38 | Toluene | B | 30.40±2.15b | 48.55±3.43a | 18.90±1.34c | 22.51±1.59b | 39.12±2.77a | 14.98±1.06c |
| 39 | p-Xylene | B | 5.02±0.35b | 8.32±0.59a | 6.14±0.43b | 5.81±0.41b | 12.29±0.87a | 3.63±0.26c |
| 40 | 1,3-Dimethyl benzene | B | 300.5±21.25b | 443.09±31.33a | 246.61±17.44b | 322.98±22.84b | 562.59±39.78a | 241.69±17.09b |
| 41 | o-Xylene | B | 5.86±0.41b | 10.34±0.73a | 4.51±0.32b | 6.17±0.44b | 12.16±0.86a | 3.91±0.28c |
| 42 | Styrene | A | 4.48±0.32b | 6.45±0.46a | 3.83±0.27b | 7.20±0.51b | 20.97±1.48a | 3.41±0.24c |
| 43 | p-Cymene | A | ND | ND | ND | 3.39±0.24a | ND | ND |
| 44 | o-Cymene | B | ND | ND | ND | 3.39±0.24a | ND | ND |
| 45 | Naphthalene | A | 1.43±0.10b | 2.10±0.15a | 1.12±0.08b | 4.20±0.30b | 10.54±0.74a | 2.85±0.20b |
| 46 | 2-Methyl-naphthalene | B | ND | ND | ND | 0.63±0.04b | 2.16±0.15a | ND |
|  | Subtotal |  | 347.68±24.58b | 518.85±36.69a | 281.12±19.88b | 376.27±26.61b | 659.83±46.66a | 270.46±19.12c |
|  | Subtotal % |  | 3.15 | 2.82 | 6.26 | 5.24 | 3.70 | 5.85 |
|  | **Total C6/C9 Compounds** | |  |  |  |  |  |  |
| 47 | Hexanal | A | 2158.32±152.62b | 3171.96±224.29a | 3039.76±214.94a | 1782.44±126.04b | 2572.82±181.93b | 3585.85±253.56a |
| 48 | 2-Hexanol | B | ND | 2.30±0.16a | ND | 2.67±0.19a | 2.56±0.18a | ND |
| 49 | (E)-2-Hexenal | A | 1098.87±77.70c | 4235.56±299.50a | 2365.22±167.25b | 1793.80±126.84b | 4720.98±333.82a | 2395.09±169.36b |
| 50 | (E)-3-Hexenyl acetate | B | ND | ND | ND | ND | ND | 0.74±0.05a |
| 51 | 1-Hexanol | A | 137.17±9.70a | 114.58±8.10a | 113.96±8.06a | 178.86±12.65a | 200.44±14.17a | 234.87±16.61a |
| 52 | (E)-3-Hexenol | A | ND | 5.13±0.36a | 5.03±0.36a | 5.41±0.38a | 5.56±0.39a | 5.31±0.38a |
| 53 | (Z)-3-Hexenol | A | 127.34±9.00a | 116.75±8.26a | 115.53±8.17a | 113.41±8.02b | 113.76±8.04b | 192.2±13.59a |
| 54 | (E)-2-Hexenol | A | 85.52±6.05a | 55.40±3.92b | 74.54±5.27a | 154.94±10.96a | 163.33±11.55a | 201.43±14.24a |
| 55 | (Z)-2-Hexenol | A | 193.84±13.71a | 123.71±8.75b | 168.88±11.94a | 351.47±24.85a | 377.09±26.66a | 457.64±32.36a |
| 56 | 2,6-Nonadienal, (E,Z)- | B | 4.08±0.29a | 4.17±0.29a | 3.56±0.25a | 4.97±0.35a | 5.09±0.36a | 4.67±0.33a |
|  | Subtotal |  | 3805.13±269.06c | 7829.56±553.63a | 5886.49±416.24b | 4387.97±310.28b | 8161.64±577.12a | 7077.79±500.48a |
|  | Subtotal % |  | 75.72 | 69.11 | 68.47 | 79.12 | 77.41 | 68.17 |
|  | **Total esters** | |  |  |  |  |  |  |
| 57 | Ethyl Acetate | A | 71.34±5.04b | 97.23±6.88a | 47.16±3.34c | 14.55±1.03a | 17.89±1.26a | 19.35±1.37a |
| 58 | n-Butyl acetate | B | 1.63±0.12a | ND | ND | ND | ND | ND |
| 59 | Butyl 2-propenoate | B | 1.95±0.14c | 11.99±0.85a | 4.16±0.29b | ND | ND | 0.18±0.01a |
| 60 | Ethyl hexanoate | A | 4.33±0.31a | 4.41±0.31a | 3.56±0.25a | 2.52±0.18a | 2.56±0.18a | ND |
| 61 | Ethyl isohexanoate | B | 4.33±0.31a | 4.41±0.31a | 3.54±0.25a | ND | 2.56±0.18a | 2.47±0.17a |
| 62 | Hexyl acetate | A | 1.06±0.07a | ND | ND | ND | 0.99±0.07a | ND |
| 63 | 2-Hexenoic acid, methyl ester, (E)- | B | ND | ND | ND | ND | 1.86±0.13a | ND |
| 64 | Methyl salicylate | | 0.54±0.04a | ND | ND | 6.89±0.49a | 5.95±0.42a | ND |
|  | Subtotal |  | 85.16±6.02b | 118.04±8.35a | 58.43±4.13c | 23.96±1.69b | 31.80±2.25a | 22.00±1.56b |
|  | Subtotal % |  | 0.27 | 0.48 | 1.53 | 1.19 | 0.77 | 0.37 |
|  | **Total norisoprenoids** | |  |  |  |  |  |  |
| 65 | 6-Methyl-5-heptene-2-one | A | 37.60±2.66a | 31.75±2.25a | 31.72±2.24a | 48.65±3.44a | 53.89±3.81a | 62.97±4.45a |
| 66 | (E)-β-Damascenone | A | 10.67±0.75b | 19.13±1.35a | 13.07±0.92b | 15.08±1.07b | 20.63±1.46a | 1.14±0.08c |
| 67 | β-Ionone | B | 23.82±1.68a | 23.10±1.63a | 19.34±1.37a | 19.41±1.37a | 21.18±1.50a | 23.61±1.67a |
| 68 | Geranylacetone | A | ND | ND | 1.28±0.09 | 2.90±0.20a | 3.15±0.22a | 3.41±0.24a |
|  | Subtotal |  | 72.09±5.10a | 73.98±5.23a | 65.41±4.63a | 86.04±6.08a | 98.85±6.99a | 91.13±6.44a |
|  | Subtotal % |  | 1.47 | 2.00 | 1.30 | 0.75 | 0.86 | 1.34 |
|  | **Total terpenoids** | |  |  |  |  |  |  |
| 69 | Camphor | C | 0.02±0.00a | 0.03±0.00a | 0.02±0.00a | 0.01±0.00a | 0.01±0.00a | 0.01±0.00a |
| 70 | Linalool | A | ND | ND | ND | ND | 1.75±0.12a | ND |
| 71 | β-Citronellol | B | ND | ND | 1.35±0.10a | 2.03±0.14a | 2.40±0.17a | 0.21±0.01a |
| 72 | Calacorene | B | 10.52±0.74a | 4.71±0.33b | 9.30±0.66a | 45.11±3.19a | 40.62±2.87a | 11.42±0.81b |
| 73 | Geraniol | A | 66.14±4.68b | 115.94±8.20a | 80.26±5.68b | 92.09±6.51b | 124.79±8.82a | 12.31±0.87c |
| 74 | Cadalene |  | 0.01±0.00a | ND | 0.01±0.00a | 0.05±0.00a | 0.06±0.00a | 0.02±0.00b |
|  | Subtotal |  | 76.70±5.42b | 120.67±8.55a | 90.95±6.43b | 139.29±9.85a | 169.63±11.99a | 23.98±1.70b |
|  | Subtotal % |  | 4.39 | 6.50 | 1.38 | 1.22 | 1.20 | 2.16 |
|  | **Total volatile phenols** | |  |  |  |  |  |  |
| 75 | Phenol, 2,5-bis(1,1-dimethylethyl)- | C | 1.84±0.13a | 0.28±0.02c | 0.90±0.06b | 1.38±0.10b | 1.07±0.08b | 1.77±0.12a |
|  | Subtotal |  | 1.84±0.13a | 0.28±0.02c | 0.90±0.06b | 1.38±0.10b | 1.07±0.08b | 1.77±0.12a |
|  | Subtotal % |  | 0.01 | 0.02 | 0.03 | 0.00 | 0.01 | 0.02 |
|  | **Others** |  |  |  |  |  |  |  |
| 76 | 2-Ethylfuran | B | 7.23±0.51a | 7.71±0.55a | 7.58±0.54a | 7.70±0.54a | 8.02±0.57a | 7.68±0.54a |
| 77 | Diacetyl | B | ND | ND | ND | ND | 153.78±10.87a | 92.81±6.56b |
| 78 | Eucalyptol | B | 7.18±0.51a | 7.15±0.51a | 7.05±0.50a | 7.52±0.53a | 6.81±0.48a | 6.73±0.48a |
| 79 | Trans-2-(2-Pentenyl)furan | B | ND | 75.62±5.35a | ND | 76.74±5.43a | ND | ND |
|  | Subtotal |  | 14.41±1.02b | 90.48±6.40a | 14.63±1.03b | 91.96±6.50b | 168.61±11.92a | 107.21±7.58b |
|  | Subtotal % |  | 0.16 | 0.11 | 0.26 | 0.91 | 0.19 | 1.43 |
|  | Sum |  | 5557.34±392.96b | 9895.95±699.76a | 7604.68±537.73ab | 6436.87±455.16b | 10938.33±773.46a | 8764.04±619.71ab |

^1^ Reliability of the identification proposal: A, identified, mass spectrum and RI agreed with standards; B and C, tentatively identified, mass spectrum and RI agreed with NIST 11 MS database and literature data.

^2^ The concentration of these compounds expressed as relative areas (to 4-methyl-2-pentanol).

^3^ Tukey’s HSD test, different letters indicate significant differences at P < 0.05.

^4^ ND, not detected.
